# Toxic Effects of Ethyl Cinnamate on the Photosynthesis and Physiological Characteristics of *Chlorella vulgaris* Based on Chlorophyll Fluorescence and Flow Cytometry Analysis

**DOI:** 10.1155/2015/107823

**Published:** 2015-05-25

**Authors:** Yang Jiao, Hui-Ling Ouyang, Yu-Jiao Jiang, Xiang-Zhen Kong, Wei He, Wen-Xiu Liu, Bin Yang, Fu-Liu Xu

**Affiliations:** MOE Laboratory for Earth Surface Processes, College of Urban & Environmental Sciences, Peking University, Beijing 100871, China

## Abstract

The toxic effects of ethyl cinnamate on the photosynthetic and physiological characteristics of *Chlorella vulgaris* were studied based on chlorophyll fluorescence and flow cytometry analysis. Parameters, including biomass, *F*_*v*_/*F*_*m*_ (maximal photochemical efficiency of PSII), Ф_PSII_ (actual photochemical efficiency of PSII in the light), FDA, and PI staining fluorescence, were measured. The results showed the following: (1) The inhibition on biomass increased as the exposure concentration increased. 1 mg/L ethyl cinnamate was sufficient to reduce the total biomass of *C. vulgaris*. The 48-h and 72-h EC50 values were 2.07 mg/L (1.94–2.20) and 1.89 mg/L (1.82–1.97). (2) After 24 h of exposure to 2–4 mg/L ethyl cinnamate, the photosynthesis of *C. vulgaris* almost ceased, manifesting in Ф_PSII_ being close to zero. After 72 h of exposure to 4 mg/L ethyl cinnamate, the *F*_*v*_/*F*_*m*_ of *C. vulgaris* dropped to zero. (3) Ethyl cinnamate also affected the cellular physiology of *C. vulgaris*, but these effects resulted in the inhibition of cell yield rather than cell death. Exposure to ethyl cinnamate resulted in decreased esterase activities in *C. vulgaris*, increased average cell size, and altered intensities of chlorophyll a fluorescence. Overall, esterase activity was the most sensitive variable.

## 1. Introduction

Algae are the main primary producers in aquatic ecosystems and are often used as environmental quality indicators [1] and experimental species for aquatic ecotoxicological research [2, 3]. Algal bloom is an important cause of the degradation of aquatic ecosystems in eutrophic water bodies [4]. Traditional chemical methods can effectively remove algae but can also cause secondary pollutions [5]. Meanwhile, traditional physical technology and biotechnology for bloom mitigation also have some disadvantages, such as long operating time, operating difficulty, and high costs [6]. In recent years, the use of allelochemicals to inhibit algal blooms has become a cost-effective method with broad application prospects [7].

Allelopathy was first introduced by Molisch in 1937 and it means that creatures, including microorganisms, exert a favorable or unfavorable influence on others through biochemical substances. Rice constrained allelopathy in his definition in 1979, holding the view that allelopathy merely meant that plants have adverse effects on other plants and the biochemical substances were released by organisms [8]. Studies have shown that allelopathy is ubiquitous in aquatic ecosystems and that almost all primary producers can produce and release allelochemicals [9]. For practical applications, there are generally three methods to inhibit algae using allelopathy of plants. (I) Cultivate aquatic plants in water and utilize the substances that are released by plants to inhibit algae. (II) Add dead plants into the water and utilize the substances that are released during the rot process to inhibit algae. (III) Add allelochemicals that have been extracted from plants into water [6].

In recent years, research on the utilization of allelochemicals to inhibit algae has become increasingly popular. Various allelochemicals have been extracted and identified, such as sesquiterpene lactones, organic acids, and phenols. Among these allelochemicals, phenolic acids (such as ethyl cinnamate) have strong activities [8–11]. Allelochemicals could affect many photosynthetic and physiological characteristics of algae, such as cell membrane permeability, reproduction, photosynthesis and respiration, protein synthesis, and resistance to diseases [12–20]. Allelochemicals affect algae selectively; therefore, different species of algae have varied inhibition responses [21–24].

Currently, research on allelopathy mainly focuses on aquatic plants and rarely on terrestrial plants, especially xylophyta. Compared with hydrophytes, terrestrial plants are more widely distributed. In addition, terrestrial plants are easy to cultivate, break, and process. Many species of herbage and xylophyta are rich in allelochemicals and, as a consequence, have better prospects in inhibiting algal blooms and the emergency response to algal blooms [25]. The allelochemicals that are produced and released by terrestrial plants can be washed from land into water by rain and interact with organisms in the water. Therefore, research on the allelochemicals, such as ethyl cinnamate, that are produced by terrestrial plants has more practical significance in inhibition of algae. However, current research on the effects of cinnamic acid and its derivatives on algal inhibition is still rare and mostly utilizes traditional methods of algal toxicology. Relevant research mainly focuses on the algal reproduction, the activities of algal antioxidant enzymes, the lipid peroxidation of cell membrane, and the interaction with nutrients such as N and K [26–29]. However, these traditional methods cannot distinguish between dead and living cells during cultivation, potentially causing variations that deviate from the real results [30]. In addition, traditional research methods of algal toxicity focus more on the toxicity of substances and less on the response of other algal physiological processes, such as photosynthesis. It is impossible to understand the mechanism of allelopathy comprehensively.

Chlorophyll fluorescence analysis and flow cytometry are new methods that have been used to study the photosynthetic toxicity and cytotoxicity of heavy metals and other pollutants on algae in recent years [2, 3]. Chlorophyll fluorescence analysis is based on the photosynthetic theory, using chlorophyll fluorescence in vivo as a probe to study the photosynthetic physiological state of organisms under the influences of various external factors [31]. Chlorophyll fluorescence analysis is rapid and sensitive, and it can also detect the photosynthetic toxicity with no damage to algae [31–34]. Flow cytometry (FCM), an automated cell or biological particles analysis technique, is capable of rapid multiple parameter detections of a single cell and has been extensively used in algal toxicity studies in recent years [35–37]. Compared with traditional methods, flow cytometry is conducive to obtaining algal responses under stress conditions on cellular level. The application of flow cytometry allows for algal toxicological research on cellular level, and this technique contributes to understanding the action mechanism of toxins.

In this study, experiments were performed to study the toxic effects of ethyl cinnamate on* Chlorella vulgaris*, a dominant species of green algae. This study, based on traditional algal toxicological research methods, used chlorophyll fluorescence analysis and flow cytometry to study how ethyl cinnamate affected the growth, photosynthesis, and cell physiology of* C. vulgaris*, in order to understand the effects and the action mechanism of allelochemicals further.

## 2. Materials and Methods

### 2.1. Experimental Materials

#### 2.1.1. Algal Strain

In this study,* Chlorella vulgaris* was selected as algal material for its two advantages.* C. vulgaris* requires relatively simple growth conditions and has a strong environmental tolerance and a high reproductive rate. In addition, many domestic and overseas researchers have also selected* C. vulgaris* as test algal material [38], which makes comparing results convenient.* C. vulgaris* was purchased from the Freshwater Algae Culture Collection of the Institute of Hydrobiology, Chinese Academy of Sciences.

#### 2.1.2. Chemical Materials

Ethyl cinnamate (99.1% purity) was purchased from Alfa Aesar (America) and used in exposure experiments of* Chlorella vulgaris*. Dimethyl sulfoxide (analytical grade) was purchased from Beijing Chemical Works (China) for the solubilization of ethyl cinnamate. Main components in BG11 medium were all purchased from Sinopharm Chemical Reagent Co., Ltd. (China) for cultivation of* C. vulgaris*. Fluorescein diacetate (FDA) was purchased from Sigma (America) for staining algal cells in flow cytometric analysis. Acetone was purchased from Beijing Chemical Works (China) for the solubilization of FDA. Propidium iodide (PI) was purchased from Sigma (America) for staining algal cells in flow cytometric analysis.

### 2.2. Experimental Methods

#### 2.2.1. Experimental Conditions

*(1) Algal Culture Conditions*.* Chlorella vulgaris* was cultivated in BG11 medium at 24 ± 1°C with a cycle of light (14 h, 4000 Lux) and dark (10 h, 0 Lux) in a GXZ-280B illumination cultivation cabinet (China).* C. vulgaris* was cultivated statically and was not aerated during the cultivation. The cultures were shaken two to three times daily and their positions were changed randomly.

*(2) Exposure Experiments*.* Chlorella vulgaris* was cultivated in 50 mL BG11 medium in 150-mL Erlenmeyer flasks (the initial cell density of* C. vulgaris* was approximately 2 × 10^6^ cells/mL).* C. vulgaris* was exposed to ethyl cinnamate at final total concentrations of 0.5 mg/L, 1 mg/L, 2 mg/L, 3 mg/L, and 4 mg/L for 96 h. In the experiment, 0.1 mL dimethyl sulfoxide was added to each sample (50 mL in total) for the solubilization of ethyl cinnamate. Preliminary results showed that 0.2% dimethyl sulfoxide (0.1 mL in 50 mL medium) had no significant impact on the photosynthetic and physiological characteristics in* C. vulgaris*. Exposure tests and controls were set up in triplicate. The biomass, chlorophyll fluorescence, and flow cytometry were measured every 24 h during the experiments.

#### 2.2.2. Measurements of the Indicators

*(1) Biomass Analysis*. Optical density of algal samples at 450 nm correlated with algal cell density determined by hemocytometer (cell density (10^6^ cells/mL) = 27.6 × OD450 and *R* ^2^ = 0.9994). Therefore, biomass was obtained by measuring the optical density of algal samples at 450 nm using a microplate reader (Model-680, Bio-Rad). The growth inhibition rate (*P*_1_) of* Chlorella vulgaris* was calculated using

(1) $$\begin{array}{c} P_{1}=\frac{N_{tcontrol}-N_{ttreatment}}{N_{tcontrol}-N_{0}}. \end{array}$$

In formula (1), *N*_*t*control_ and *N*_*t*treatment_ represent the optical density of the blank control and the treatment at time *t*. *N*_0_ represents the initial optical density. The medium effective concentration (EC50) inhibiting 50% the biomass at time *t* was calculated by fitting *P*_1_ and ethyl cinnamate concentration with a linear regression.

*(2) Chlorophyll Fluorescence Analysis*. In this study, chlorophyll fluorescence was used to measure the photosynthetic activity of* Chlorella vulgaris*. Values of chlorophyll fluorescence are related with the switch state of reaction center of PSII. Chlorophyll fluorescence cannot be detected from dead cells. Thus, chlorophyll fluorescence can be used as a probe to monitor the photosynthesis of* C. vulgaris*.

The parameters, including *F*_0_ (minimal fluorescence), *F*_*m*_ (maximal fluorescence), and *Ф*_PSII_ (actual photochemical efficiency of PSII in the light), were measured by MAXI-Imaging-PAM (Walz, Germany) after adaptation in the dark for 25 min. The *F*_*v*_/*F*_*m*_ or (*F*_*m*_ − *F*_0_)/*F*_*m*_ (maximal photochemical efficiency of PSII) was obtained by calculation. What is noteworthy is that when algal cells are in normal state, the number of cells has little effect on *F*_*v*_/*F*_*m*_ and *Ф*_PSII_.

The parameter, ETR (photosynthetic electron transport rate), can be calculated automatically by the instrument according to the formula as follows:

(2) $$\begin{array}{c} \text{ETR}=\text{Yield}\times 0.84\times 0.5\times \text{PAR}. \end{array}$$

Yield is actual photochemical efficiency of PSII in the light (*Ф*_PSII_); 0.84 is empirical constant (absorption rate of light from blade); 0.5 is due to photosynthetic system including two centers and electron transport in one center needing two photons; PAR is photosynthetically active radiation.

*(3) Flow Cytometric Analysis*. The flow cytometer BD FACSCalibur was used to measure the parameters of algal cells. The negative control (heat-treated cells at 100°C for 10 min) was set before the experiments. Before the measurement, the voltage level of each detection channel was adjusted according to the fluorescent intensity of the heat-treated cells and normal cells, to make the fluorescent intensity of the normal algal cells in the middle level while reserving enough space for affected cells. And during the measurement, the voltage level was constant.

Fluorescein diacetate (FDA) was used to assess the esterase activities during the experiments [30, 35, 37]. The FDA (dissolved in acetone to a final concentration of 25 *μ*mol/L) stained the algal cells for 8 min in the dark, and then the fluorescence in the FL1 channel (515–545 nm) was measured. An acetone control was added to eliminate effects resulting from acetone. The results demonstrated that acetone had no effect on the esterase activities of* Chlorella vulgaris*.

Propidium iodide (PI) was used to assess the integrity of the cell membrane [36, 37, 39]. The PI (final concentration 10 *μ*mol/L) stained the algal cells for 15 min in the dark, and then the fluorescence in the FL2 channel (564–606 nm) was measured.

Cellular autofluorescence (chlorophyll a fluorescence) was detected in the FL3 channel (>650 nm) while measuring the FDA and PI staining fluorescence.

### 2.3. Data Analysis

Using Excel 2010 for statistical calculations, the results were expressed as the triplicate arithmetic mean ± the standard deviation. ANOVA and two-factor analysis of variance were performed by SPSS 20.0. Significant difference levels were set to 0.05, *p* > 0.05, and *p* < 0.05, respectively, representing no significant difference and significant difference.

Summit 5.0 software was used for the data that was obtained by the flow cytometry, the fluorescent analysis of algal cells using histograms and the scatter plots. The fluorescent intensity in channel FL3 could help distinguish between algal cells and impurities to reduce the interference. To analyze the data, the Gate should be set in accordance with the fluorescent intensity in channel FL3, and the fluorescent intensity of the sample in the Gate could be used to calculate the mean fluorescent intensity of the algal cells (MFI) and the ratio of normal algal cells.

## 3. Results and Discussion

### 3.1. Growth Inhibition

**Figure 1:**
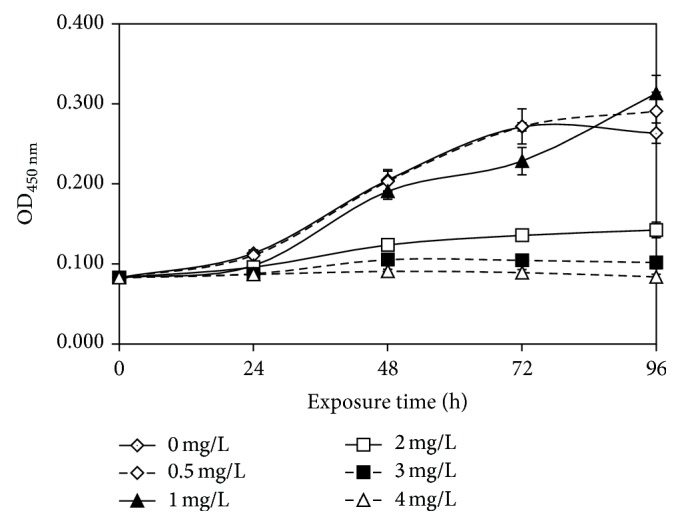
The effects of ethyl cinnamate on the biomass of* Chlorella vulgaris*. (The values represent the means ± 1 standard deviation and *n* = 3.)

The effects of ethyl cinnamate on the growth of* Chlorella vulgaris* are shown in Figure 1. After exposure to 0.5 mg/L ethyl cinnamate and a blank control for 96 h, the biomass decreased compared with that after exposure for 72 h. However, under the 1 mg/L ethyl cinnamate treatment, the biomass increased sustainably. The blank control and the 0.5 mg/L ethyl cinnamate treatment may have extra interference, possibly causing the biomass of* C. vulgaris* to be stagnant or even suppressed. As a consequence, after 72 h, 0.5 mg/L ethyl cinnamate had no significant effect on the biomass of* C. vulgaris* (*p* > 0.05). However, with the increasing concentrations of ethyl cinnamate, the degree of algal cell yield inhibition gradually increased. The exposure concentrations, exposure duration, and interaction of the two factors significantly influenced the biomass of* C. vulgaris* (*p* < 0.05). The 48-h and 72-h EC50 of ethyl cinnamate were 2.07 mg/L (1.94–2.20) and 1.89 mg/L (1.82–1.97).

The study results of Pinheiro et al. [40] on the effects of microcystin-LR (MC-LR) and cylindrospermopsin (CYN) (they are also regarded by some researchers as allelopathic substances) on* C. vulgaris* indicated that MC-LR and CYN at environmentally occurring concentrations were unable to affect negatively growth of* C. vulgaris*. This could be due to the fact that these molecules played roles other than allelopathy in natural ecosystems or the selectivity of allelochemicals.

### 3.2. Photosynthetic Toxicity

**Figure 2:**
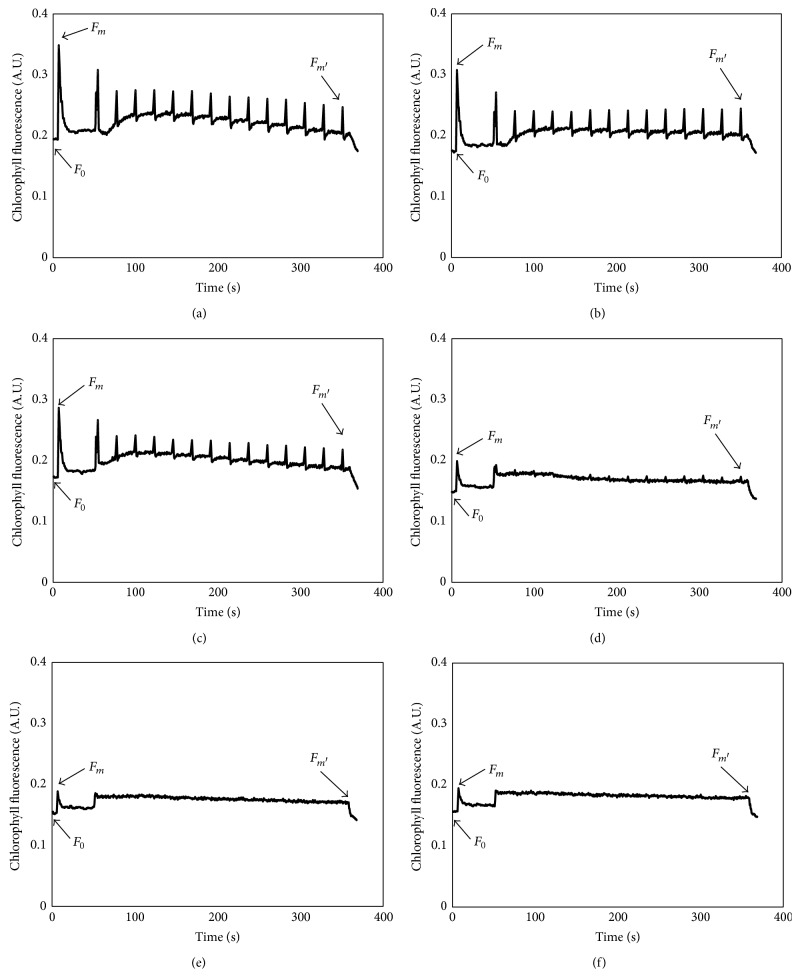
The photoinduced curves of* Chlorella vulgaris* after 24 h of exposure to ethyl cinnamate. ((a) Blank control, (b) 0.5 mg/L treatment, (c) 1 mg/L treatment, (d) 2 mg/L treatment, (e) 3 mg/L treatment, and (f) 4 mg/L treatment.)

The photoinduced curves of* Chlorella vulgaris* after exposure to ethyl cinnamate for 24 h are shown in Figure 2. After exposure to the 0.5 mg/L ethyl cinnamate for 24 h, the *F*_0_, *F*_*m*_, and *F*_*m*′_ decreased by 4.1%, 6.9%, and 0.9%, respectively (Figure 2(b)). The decrease degree of chlorophyll fluorescence increased with the concentration of ethyl cinnamate. When the concentration of ethyl cinnamate reached 2 mg/L, the *F*_0_, *F*_*m*_, and *F*_*m*′_ decreased by 12.1%, 35.6%, and 23.4%, respectively. When the concentrations of ethyl cinnamate were more than 2 mg/L, the chlorophyll fluorescence of* C. vulgaris* was significantly inhibited. Although there was potential for photosynthesis, the actual photosynthetic activities were low, with fluctuations of chlorophyll fluorescence curves close to 0 for specific performance (Figures 2(e) and 2(f)).

**Figure 3:**
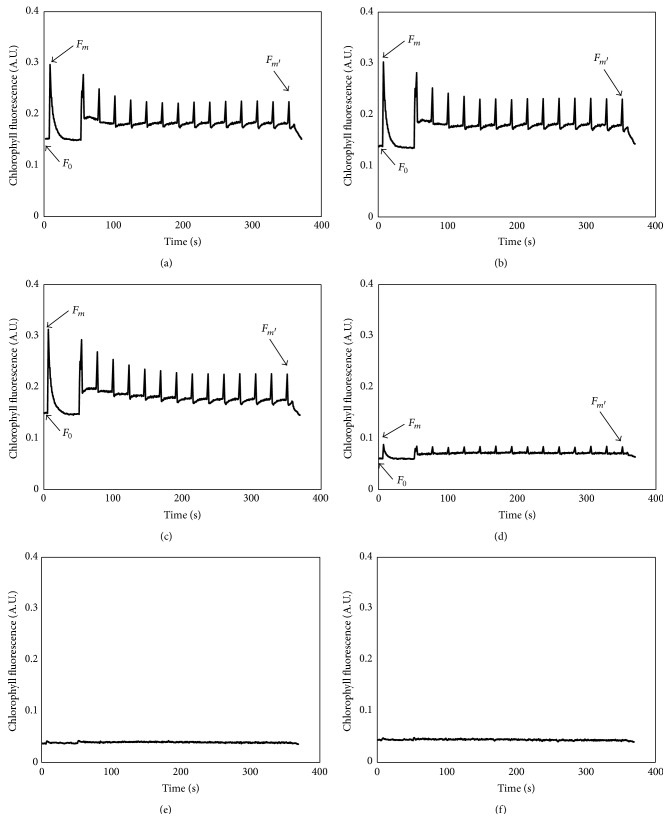
The photoinduced curves of* Chlorella vulgaris* after 96 h of exposure to ethyl cinnamate. ((a) Blank control, (b) 0.5 mg/L treatment, (c) 1 mg/L treatment, (d) 2 mg/L treatment, (e) 3 mg/L treatment, and (f) 4 mg/L treatment.)

The photoinduced curves of* C. vulgaris* under the blank control and ethyl cinnamate treatment after 96 h are shown in Figure 3. 0.5 mg/L and 1 mg/L ethyl cinnamate had little impact on the chlorophyll fluorescence of* C. vulgaris*. Compared with those of blank control, the *F*_0_, *F*_*m*_, and *F*_*m*′_ of* C. vulgaris* exposed to 0.5 mg/L ethyl cinnamate decreased by 4.3%, 0.2%, and 3.3%, respectively. Although the *F*_0_ of* C. vulgaris* under 1 mg/L treatments decreased by 3.2%, the *F*_*m*_ and *F*_*m*′_ increased by 4.9% and 1.3%, respectively. The 2 mg/L ethyl cinnamate affected the chlorophyll fluorescence of* C. vulgaris* significantly, for *F*_0_, *F*_*m*_, and *F*_*m*′_ decreasing by 63.5%, 73.0%, and 67.1%, respectively. The photoinduced curves of* C. vulgaris* were nearly linear under the 3 mg/L and 4 mg/L treatments, indicating that photosynthesis was completely inhibited in these two treatment groups (Figures 3(e) and 3(f)).

**Figure 4:**
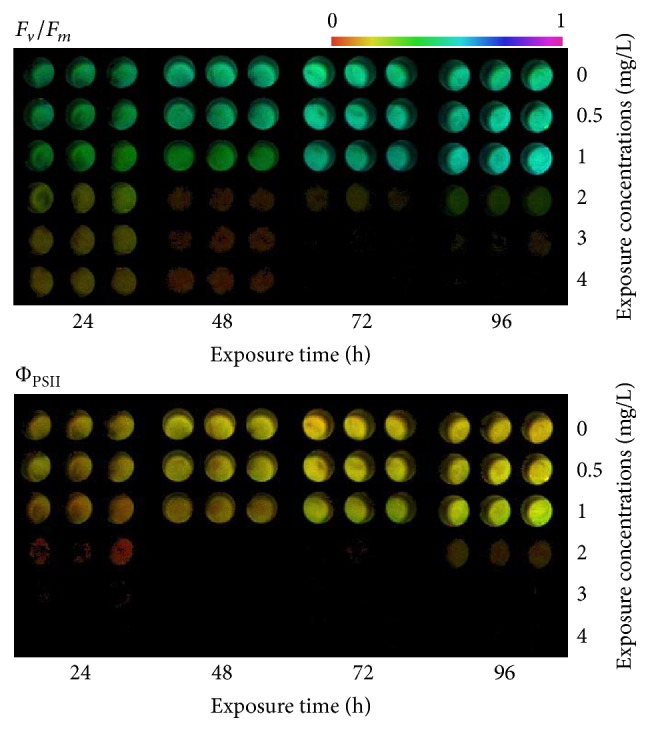
The fluorescent imaging of* C. vulgaris* under the stress of ethyl cinnamate.

**Figure 5:**
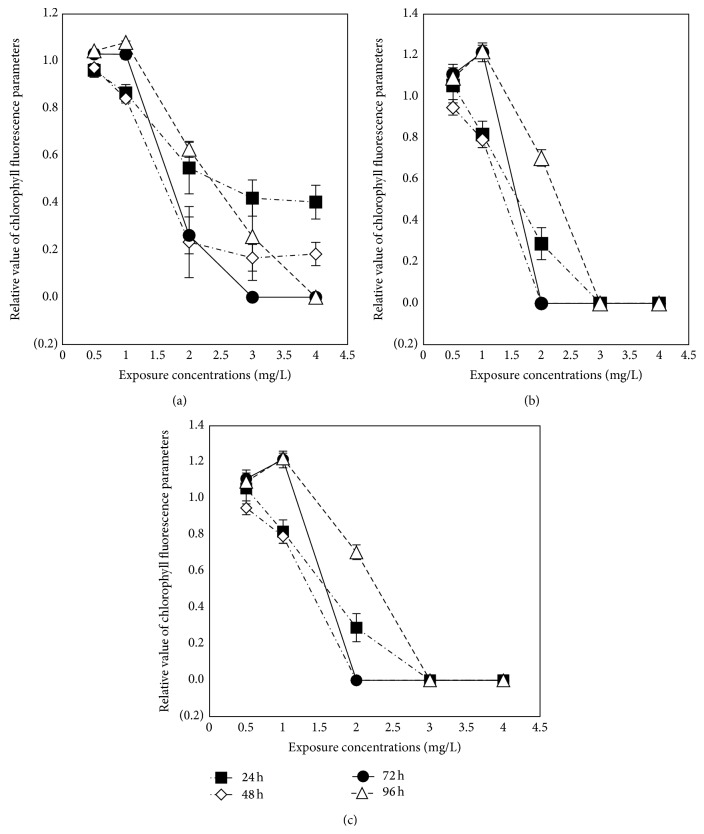
The effects of ethyl cinnamate on the *F*_*v*_/*F*_*m*_, *Ф*_PSII_, and ETR of* C. vulgaris*. ((a) *F*_*v*_/*F*_*m*_; (b) *Ф*_PSII_; (c) ETR.) (The relative value of chlorophyll fluorescence parameters: treatment-to-blank control ratio of chlorophyll fluorescence parameters.)

The *F*_*v*_/*F*_*m*_ (maximal photochemical efficiency of PSII), *Ф*_PSII_ (actual photochemical efficiency of PSII in the light), ETR (photosynthetic electron transport rate), and chlorophyll a fluorescence were used to manifest the photosynthetic toxicity of ethyl cinnamate on* C. vulgaris*. Values of maximum photochemical efficiency (*F*_*v*_/*F*_*m*_) reflect the potential quantum efficiency of PSII and the decline in values will be seen when plants are under stress [31]. As a consequence, *F*_*v*_/*F*_*m*_ could be a sensitive indicator for photosynthesis. Actual photochemical efficiency measures the proportion of the light absorbed by chlorophyll associated with PSII that is used in photochemistry, also getting lower when plants are under stress [31]. In the experiment, the chlorophyll fluorescent imaging of* C. vulgaris* under the stress of ethyl cinnamate is shown in Figure 4 and the effects of ethyl cinnamate on the *F*_*v*_/*F*_*m*_, *Ф*_PSII_, and ETR of* C. vulgaris* are shown in Figure 5.

0.5 mg/L ethyl cinnamate had little impact on the *F*_*v*_/*F*_*m*_ of* C. vulgaris*. Although 1 mg/L ethyl cinnamate affected the *F*_*v*_/*F*_*m*_ in the first 48 h, there was no difference between the *F*_*v*_/*F*_*m*_ of blank control and that of the ethyl cinnamate treatment group after exposure for 72 h and 96 h. 2–4 mg/L ethyl cinnamate significantly affected the *F*_*v*_/*F*_*m*_ of* C. vulgaris*, and the effects increased with the exposure concentration and time. After exposure to 4 mg/L ethyl cinnamate for 72 h and 96 h, the photosynthesis of* C. vulgaris* was completely (100%) inhibited.

Compared with the *F*_*v*_/*F*_*m*_, ethyl cinnamate inhibited the *Ф*_PSII_ and ETR of* C. vulgaris* much more. After exposure to 2 mg/L ethyl cinnamate for 24 h,* C. vulgaris* barely performed photosynthesis, with a slight recovery occurring after 96 h. The *Ф*_PSII_ and ETR of* C. vulgaris* under the 3 mg/L and 4 mg/L ethyl cinnamate treatments were 0 during the entire exposure process.

**Figure 6:**
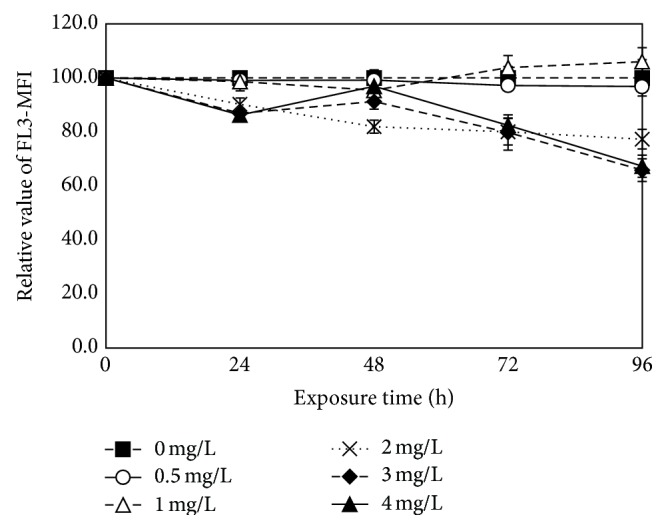
The effects of ethyl cinnamate on the fluorescence of chlorophyll a.

Chlorophyll a fluorescence reflects chlorophyll a concentration. The decrease of values of chlorophyll a fluorescence often indicates that the photosynthesis of plants is inhibited. This parameter could also be a sensitive indicator for photosynthesis. Regarding the ratio of normal fluorescent cells, 0.5–4 mg/L ethyl cinnamate had no significant impact on the FL3 fluorescence (the autofluorescence of chlorophyll a) (*p* > 0.05) but affected the mean FL3 fluorescence (the mean of FL3 fluorescence value for all tested algal cells) in the different treatment groups, as shown in Figure 6. Studies have shown that the inhibition of the electron acceptor of PSII reaction centers led to an increase in the chlorophyll a fluorescence [41, 42], while the inhibition of the electronic supply resulted in a decrease [41]. In this study, chlorophyll content did not reduce significantly (according to the measurement results of the fluorescence of chlorophyll a) and the photosynthetic activities were inhibited. Based on the results, it was speculated that ethyl cinnamate inhibited the photosynthetic rates of* C. vulgaris* mainly through electron transfer chain rather than the structure of the chloroplasts.

The study results of Gao et al. [26] on the effects of ethyl cinnamate on* Chlorella pyrenoidosa* revealed a decrease of chlorophyll a and inhibition of growth, consistent with the results in this study. The study results of Brückner et al. [43] on the effects of ragweed inflorescence extract on the growth of* C. vulgaris* and* Chlamydomonas* sp. showed the allelochemicals in the extract decreased chlorophyll a content of* C. vulgaris* significantly. In this study, ethyl cinnamate had no significant impact on the FL3 fluorescence but affected the mean FL3 fluorescence, consistent with the results of Brückner et al. to some extent.

### 3.3. Cellular Physiological Toxicity

**Figure 7:**
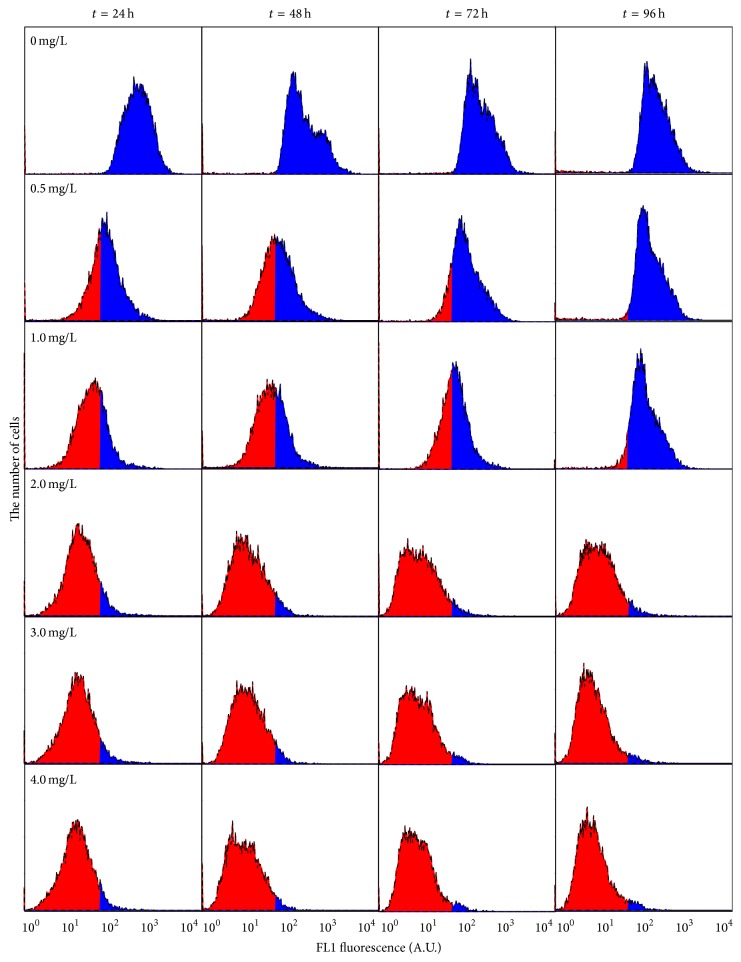
The effects of ethyl cinnamate on the FDA staining fluorescence of* Chlorella vulgaris*.

FDA and PI staining fluorescence, as well as FSC fluorescence, were measured to provide information in toxic effects of ethyl cinnamate on cellular physiology of* Chlorella vulgaris*. FDA staining fluorescence represents esterase activities of cells, and FDA only stains living cells. Thus, FDA staining fluorescence is a sensitive indicator for distinguishing between living cells and dead ones. Higher values of FDA staining fluorescence indicate higher esterase activities. Correspondingly, low values of FDA staining fluorescence mean low esterase activities; in other words, the cells are inhibited. The effects of ethyl cinnamate on the FDA staining fluorescence of* Chlorella vulgaris* are shown in Figure 7. The blue area of the image represents the normal algal cells, the area of which can be used to calculate the ratio of normal fluorescent cells. The red area demonstrates that the esterase activities of* C. vulgaris* were affected; therefore, the FDA staining fluorescence is outside the normal range. The 0.5 mg/L and 1 mg/L ethyl cinnamate caused the ratio of normal fluorescent cells to be lower than that of the blank control; nevertheless, this ratio increased with the exposure time. After exposure for 96 h, there were no prominent distinctions between the ratios of normal fluorescent cells of the blank control and that of the ethyl cinnamate treatment groups. Under the 2–4 mg/L ethyl cinnamate treatments, the ratios of algal cells of which the fluorescence was affected were much higher than normal cells. In addition, as the exposure time increased, so did the ratio of affected algal cells.

**Figure 8:**
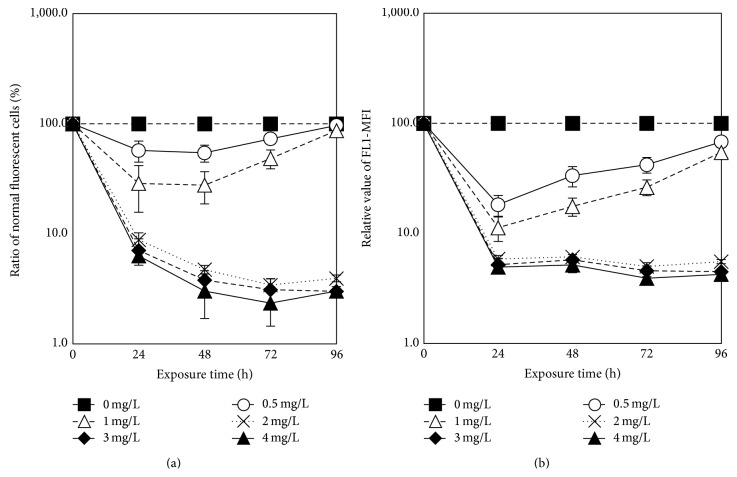
The effects of ethyl cinnamate on the esterase activities of* C. vulgaris*. ((a) The ratio of normal fluorescent cells and (b) the mean FL1 fluorescence.)

The effects of ethyl cinnamate on the esterase activities of* C. vulgaris* are shown in Figure 8. The 0.5–4 mg/L ethyl cinnamate treatments decreased the esterase activities of* C. vulgaris*, which could be observed even after 24 h, indicating that both the ratios of normal fluorescent cells and the FL1-MFI were lower than those of the blank control. In addition, the effects of ethyl cinnamate on the esterase activities of* C. vulgaris* were concentration-dependent. Under low ethyl cinnamate concentrations (0.5 and 1 mg/L), as the exposure time increased, the degree of esterase activity inhibition decreased. The ratios of normal fluorescent cells were not much different from blank control after 96 h; nevertheless, the FL1 fluorescence indicated that the esterase activities were still lower than blank control. Under high ethyl cinnamate concentrations (2, 3, and 4 mg/L), the degree of esterase activity inhibition increased with the exposure time, indicating that the ratios of normal fluorescent cells decreased continuously.

PI staining fluorescence could reflect in the integrity of the cell membrane, and PI only stains dead cells. As a result, the values of this parameter also represent mortality. High mortality means high values of PI staining fluorescence. From the results of PI staining, cells of* C. vulgaris* under treatment and blank control were in the same range, while no cells were found in the range of affected cells. This demonstrated that ethyl cinnamate had no significant effect on the integrity of the cell membrane of* C. vulgaris* (data not shown). Compared with the results in Section 3.1, it was possible that the effects of ethyl cinnamate on the growth of* C. vulgaris* were mainly the suppression of the agamogony. According to the study results of Gao et al. [26], ethyl cinnamate induced the overaccumulation of ROS and the increase of MDA, suggesting that ethyl cinnamate could lead to the damage of cell membrane system. However, according to this study, ethyl cinnamate had no significant effects on cell membrane integrity of* C. vulgaris*, demonstrating allelochemicals had diverse effects on different species of algae, in other words, selectivity. This perhaps also demonstrated* Chlorella pyrenoidosa* was more sensitive to allelochemicals.

**Figure 9:**
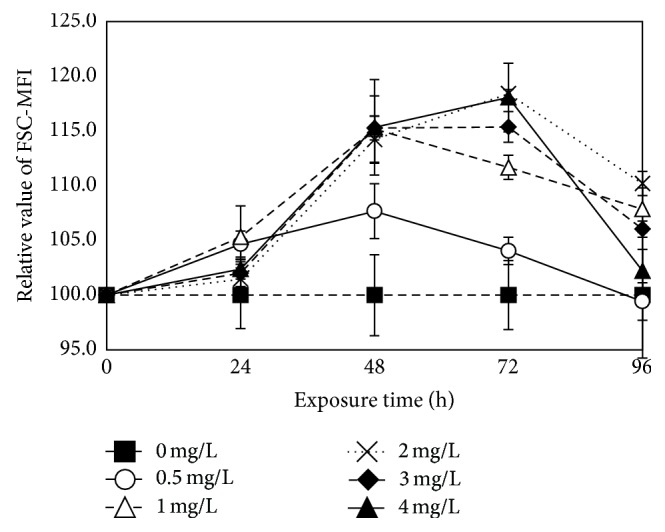
The effects of ethyl cinnamate on the size of the* C. vulgaris* cells.

The values of FSC fluorescence are relevant to the size of cells. The values of FSC fluorescence get higher with the size of cells being bigger. Ethyl cinnamate also significantly affected the size of the* C. vulgaris* cells. As shown in Figure 9, ethyl cinnamate increased the size of the* C. vulgaris* cells.

## 4. Conclusions

Ethyl cinnamate could significantly affect the growth, photosynthesis, and cellular physiology of* Chlorella vulgaris*. 1 mg/L ethyl cinnamate effectively inhibited the growth of* C. vulgaris*. The 48-h and 72-h EC50 values were 2.07 mg/L (1.94–2.20) and 1.89 mg/L (1.82–1.97), respectively. Ethyl cinnamate significantly inhibited the *F*_*v*_/*F*_*m*_ of* C. vulgaris* at concentrations of 2, 3, and 4 mg/L, with an even greater inhibition on the *Ф*_PSII_ and ETR. Based on the results, it was speculated that ethyl cinnamate inhibited the photosynthetic rate of* C. vulgaris* mainly through affecting the electron transfer chain rather than the structure of the chloroplasts. On individual cell basis, ethyl cinnamate decreased esterase activities in the cell but did not alter the integrality of cell membrane. It also resulted in increasing average cell size.
